# Predictors and Implications of Early Clinical Stability in Patients Hospitalized for Moderately Severe Community-Acquired Pneumonia

**DOI:** 10.1371/journal.pone.0157350

**Published:** 2016-06-15

**Authors:** Nicolas Garin, Garance Felix, Christian Chuard, Daniel Genné, Sebastian Carballo, Olivier Hugli, Olivier Lamy, Christophe Marti, Mathieu Nendaz, Olivier Rutschmann, Stephan Harbarth, Arnaud Perrier

**Affiliations:** 1 Division of General Internal Medicine, Geneva University Hospitals and Faculty of Medicine, Geneva, Switzerland; 2 Division of Internal Medicine Regional Hospital Riviera-Chablais, Monthey, Switzerland; 3 Division of Internal Medicine, Hôpital cantonal, Fribourg, Switzerland; 4 Division of Internal Medicine, Centre Hospitalier de Bienne, Bienne, Switzerland; 5 Department of Emergency Medicine, Centre Hospitalier Universitaire Vaudois, Lausanne, Switzerland; 6 Division of Internal Medicine, Centre Hospitalier Universitaire Vaudois, Lausanne, Switzerland; 7 Emergency Department, Geneva University Hospitals and Faculty of Medicine, Geneva, Switzerland; 8 Infection Control Programme, Geneva University Hospitals and Faculty of Medicine, Geneva, Switzerland; University of Dundee, UNITED KINGDOM

## Abstract

**Background:**

Assessment of early response to treatment is crucial for the management of community-acquired pneumonia (CAP).

**Objective:**

To describe the predictors and the outcomes of early clinical stability

**Methods:**

We did a secondary analysis of a multicentre randomized controlled trial on CAP treatment in which 580 patients hospitalized for moderately severe CAP were included. The association between demographic, clinical and biological variables available at inclusion and early clinical stability (stabilization of vital signs within 72 hours with predetermined cut-offs) was assessed by multivariate logistic regression. The association between early clinical stability and mortality, severe adverse events, and length of stay was also tested.

**Results:**

Younger age (OR 0.98, 95% CI 0.96–0.99), lower platelet count (OR per 10 G/L increment 0.96, 95% CI 0.94–0.98), lower respiratory rate (OR 0.94, 95% CI 0.90–0.97), absence of hypoxemia (OR 0.58, 95% CI 0.40–0.85), lower numbers of co-morbid conditions (OR 0.82, 95% CI 0.69–0.98) and signs or symptoms (OR 0.78, 95% CI 0.68–0.90) were significantly associated with early clinical stability. Patients with early clinical stability had lower 90-days mortality (3.4% vs. 11.9%, p<0.001), fewer admissions to the intensive care unit (2.7% vs. 8.0%, p = 0.005) and a shorter length of stay (6.0 days, IQR 4.0–10.0 vs. 10.0 days, IQR 7.0–15.0, p<0.001).

**Conclusions:**

Patients with younger age, less co-morbidity, fewer signs or symptoms, less respiratory compromise, and a lower platelet count are more likely to reach early clinical stability. Patients without early clinical stability have a worse prognosis and warrant close scrutiny.

## Introduction

Community-acquired pneumonia (CAP) is a heterogeneous disease ranging from a mild self-limiting disease to a severe infection causing respiratory failure, shock, and death. Costs of CAP in Europe are estimated to more than 10 billion per year, more than half being attributed to inpatient care.[1] Antibiotic treatment is often empirical as the responsible pathogen is known only in 30–50% of cases.[2]

Early assessment of response to treatment is an important step in CAP management as it is linked to important clinical decisions such as changing the empiric antibiotic treatment, performing new investigations, switching to oral antibiotics, or discharging the patient from the hospital. It has also been advocated as an important endpoint in clinical trials comparing different treatment regimens, [3] and can be considered as a surrogate for discharge readiness.[4] The course of CAP has been schematically described as early (within 3 days) or late (3 to 7 days) recovery, usually interpreted as response, or lack of, to therapy.[5] A first assessment of treatment response at day 3 is based on older studies suggesting that a difference in the evolution between patients treated or not with an antibiotic agent is not apparent earlier.[6] It is also a relevant time point as the results of the initial bacteriological investigations are usually available, if positive. Finally, 3 days was the median time needed to reach clinical stability according to the milestone study by Halm et al.[7] The incidence of early failure of treatment (defined as a deterioration of the clinical or radiological status) is 6 to 16%.[8,9] In addition, some patients may have no improvement in clinical signs and symptoms without meeting the criteria for failure, a situation sometimes described as non-resolving CAP.[10] In a contemporary cohort, nearly 50% of patients still had abnormal vital signs, i.e. were not clinically stable after 3 days of treatment.[11]

Clinicians facing treatment failure or lack of improvement in patients hospitalized for CAP at this time point frequently change the antibiotic regimen to cover more pathogens, or perform additional diagnostic tests. Broadening of antimicrobial spectrum after >72 hours was performed in 15.9% to 28.9% of patients included in recent cohort studies, and was mostly due to insufficient response or treatment failure.[12,13,14] Moreover, 34.8% of patients with late (more than 4 days) stability had a modification in the prescribed treatment, as compared with 14.2% of patients with earlier stability.[15] However, initial antibiotic treatment is not always inadequate in early failure or lack of improvement, as there are many alternative causes.[9,16] Rather, an inadequate inflammatory response of the host has been incriminated as the most frequent reason of early failure.[8]

The aim of this study was to determine factors independently associated with early clinical stability in patients hospitalized for CAP. Better knowledge of these factors should help clinicians to identify patients warranting closer monitoring of treatment response. A second aim was to describe the association between early clinical stability and other outcomes in CAP, including modifications of the antibiotic treatment after the initial 72 hours and severe adverse events.

## Methods

The study was approved by the Institutional Review Board of Geneva University Hospitals (Nr 06–259), the IRBs of all hospitals including patients, and the Swiss agency for drugs approval and regulation, Swissmedic (ID 2008 DR 4371). All patients provided written informed consent. We did a secondary analysis of a multicentre randomized trial that compared two antibiotic strategies (betalactam monotherapy versus betalactam-macrolide combination therapy) in patients hospitalized for moderately severe CAP (clinical trials.gov identifier:NCT00818610).[17] Definitions, inclusion and exclusion criteria, data collection, and follow-up are described in detail in the original publication. Briefly, adult patients with radiologically confirmed CAP and needing hospitalisation were included at 6 acute care hospitals in Switzerland. Main exclusion criteria were severe CAP (including need for immediate ICU admission, PSI category V, or three or more minor criteria of the ATS/IDSA 2007 rule), severe immunosuppression, and nursing home residency. Vital parameters were measured twice a day under standardized conditions. The primary outcome of the initial clinical trial was the proportion of patients with clinical stability at 7 days, defined as simultaneous normalization of the vital signs according to the following criteria: heart rate < 100 bpm, systolic blood pressure > 90 mmHg, temperature < 38.0°C, respiratory rate < 24 per min, and oxygen saturation > 90% on room air. Patients dying in the hospital were censored at the end of the study (meaning that they were counted as never reaching clinical stability). Follow-up extended up to 90 days after admission.

For the purpose of this secondary analysis, we determined clinical stability 72 hours after the start of the treatment, using the same definition. The predictor variables were selected among demographic, clinical and laboratory data available at admission. Descriptive statistics were used, with frequencies, proportions, medians with interquartile range and means with standard deviation. We used two-sided tests and a significance level of 0.05 for statistical hypothesis testing. The univariate association between early clinical stability and predictor variables, and between early clinical stability and other outcomes, was tested with a Chi square test, Fisher exact test or analysis of variance, as indicated. Variables associated with the outcome in univariate analysis with a p value <0.2 were then incorporated in a first multivariate binary logistic regression model. Severity scores (Pneumonia Severity Index (PSI)[18] and Confusion, Urea >7 mmol/l, Respiratory rate ≥30/min, low systolic(<90 mm Hg) or diastolic (≤60 mm Hg) Blood pressure), age ≥65 years (CURB-65)[19]) were not included because we expected high colinearity with other variables included. Since some of the variables used in the definition of the outcome (early clinical stability) were also included as predictors in the multivariable model, we fitted a second multivariate model, excluding predictor variables that were included in the definition of clinical stability. Post-hoc analyses adjusting for PSI and CURB-65 scores, which are widely validated prognostic tools, were done for the association between lower platelet count and early clinical stability. The minimal data set underlying the finding of this study is available as supporting information. (S1 Table)

## Results

A total of 580 patients (all patients included in the original clinical trial) were available for the analyses. One or more missing value was present in 51 (8.8%) patients, who were excluded from the multivariate analyses. Median age was 76 years (range 21–101 years), mean PSI score was 84 (risk class III), and a pathogen was identified in 180 (31.0%) patients. *Streptococcus pneumoniae* was identified in 88 (15.2%), *Legionella pneumophila* in 16 (2.8%) and *Mycoplasma pneumoniae* in 15 (2.6%) patients. Seventy-two hours after the beginning of the treatment, 293 (50.5%) patients met the criteria for clinical stability (early clinical stability).

### Factors associated with early clinical stability in univariate analysis

Age, number of co-morbid conditions and of initial signs or symptoms, respiratory rate, presence of hypoxemia, blood concentration of urea and glucose, and platelet count were all significantly associated with clinical stability in the univariate analysis, as were the PSI and CURB-65 scores. (Table 1) Early clinical stability was present in 65% of patients < 65 years, 49% of patients between 65 and 85 years, and only 35% of patients >85 years old. Early clinical stability was reached in 58% of patients without co-morbid conditions compared with 46% of patients with one or more co-morbid condition.

**Table 1:** Univariate analysis of variables associated with early clinical stability (≤ 3 days).

	Early stability (N = 293)	No stability (N = 287)	P-value
**Demography**			
**Male sex No. (%)**	172 (58.7)	161 (56.1)	0.53
**Age (median with IQR)**	73.0 (59.5–82.5)	77.0 (67.0–85.0)	<0.001
**Number of co-morbid conditions**	0.9 (1.0)	1.2 (1.1)	<0.001
**Clinical presentation**			
**Number of initial symptoms / signs**^a^	4.7 (1.3)	5.0 (1.24)	0.01
**Heart rate (‘/min)**	97 (20)	100 (20)	0.07
**Respiratory rate (‘/min)**	22.7 (5.8)	25.4 (6.0)	<0.001
**Systolic blood pressure (mmHg)**	132 (23)	135 (23)	0.06
**Diastolic blood pressure (mmHg)**	73 (14)	74 (15)	0.58
**Temperature (°C)**	37.9 (1.1)	37.9 (1.0)	0.56
**Ancillary tests**			
**Pleural effusion No. (%)**	44 (15.0)	53 (18.5)	0.32
**Hypoxemia** ^b^ **No. (%)**	119 (41.9)	172 (60.4)	<0.001
**Arterial pH**	7.44 (0.05)	7.44 (0.05)	0.23
**Arterial PO2 (kPa)**	9.7 (3.1)	9.6 (2.9)	0.80
**Urea (mmol/L)**	7.1 (4.6)	8.2 (4.6)	0.007
**Sodium (mmol/L)**	136 (4)	136 (4)	1.0
**Glucose (mmol/L)**	7.3 (2.6)	7.9 (2.8)	0.008
**Hematocrit (%)**	39.5 (4.8)	38.9 (5.2)	0.18
**Leukocytes (G/L)**	13.7 (6.3)	13.3 (6.4)	0.42
**Procalcitonin (ug/L)**	3.3 (11.0)	3.9 (15.2)	0.57
**Platelets (G/L)**	221 (91)	245 (102)	0.002
**Severity scores**			
**PSI score**	78.2 (26.7)	90.6 (21.4)	<0.001
**PSI risk class No. (%)**			
**I**	41 (14.0)	13 (4.5)	
**II**	66 (22.5)	39 (13.6)	
**III**	89 (30.4)	92 (32.1)	
**IV**	97 (33.1)	143 (49.8)	<0.001
**CURB-65 score**	1.4 (0.9)	1.8 (0.9)	<0.001
**CURB-65 score≥ 2 No. (%)**	133 (45.4)	178 (62.0)	<0.001
**Received antibiotics before admission No. (%)**	12 (4.1)	14 (4.9)	0.65
**Received combination therapy** ^c^ **No. (%)**	138 (48.1)	151 (51.5)	0.41

All numeric variables are presented as means and standard deviations if not stated otherwise.

^a^ signs and symptoms of pneumonia assessed at admission: new or increasing cough, fever (> 38.0 C), purulent sputum, pleuretic chest pain, new or increasing dyspnea, tachpnea (> 18 /min), focal auscultatory findings

^b^ Hypoxemia: oxygen saturation = <90% on room air or need for supplemental oxygen to maintain an O2 saturation > 90%

^c^ Combination therapy was amoxicillin / clavulanic acid or cefuroxime plus clarithromycine. All other patients were treated with amoxicillin / clavulanic acid or cefuroxime alone

PSI: Pneumonia Severity Index CURB-65: Confusion, Urea, Respiratory rate, Blood pressure, 65 years old.

### Factors associated with early clinical stability in multivariate analysis

In the first model, younger age, lower respiratory rate, absence of hypoxemia and lower platelet count were independently associated with early clinical stability. In the second model excluding variables incorporated in the definition of early clinical stability, younger age, lower number of co-morbid conditions, lower number of symptoms or signs, and lower platelet count were independent predictors of early clinical stability (Table 2).

**Table 2:** Multivariate analysis of variables associated with early clinical stability.

	First model		Second model	
	Odd ratio (95% C.I.)	p-value	Odd ratio (95% C.I.)	p-value
**Age (per 1-year increment)**	0.98 (0.96–0.99)	0.001	0.98 (0.96–0.99)	<0.001
**Number of co morbid conditions**	0.86 (0.71–1.04)	0.11	0.82 (0.69–0.98)	0.03
**Number of symptoms/signs**	0.86 (0.74–1.01)	0.06	0.78 (0.68–0.90)	0.001
**Respiratory rate (‘/min)**	0.94 (0.90–0.97)	<0.001		
**Hypoxemia**#	0.58 (0.40–0.85)	0.01		
**Platelets (per 10 G/L increment)**	0.96 (0.94–0.98)	<0.001	0.97 (0.95–0.99)	0.001

^#^ Hypoxemia: oxygen saturation = <90% on room air or need for supplemental oxygen

An odd ratio <1 means that patients presenting the characteristic are less likely to reach early clinical stability.

### Association of platelet count with early clinical stability

**Fig 1:**
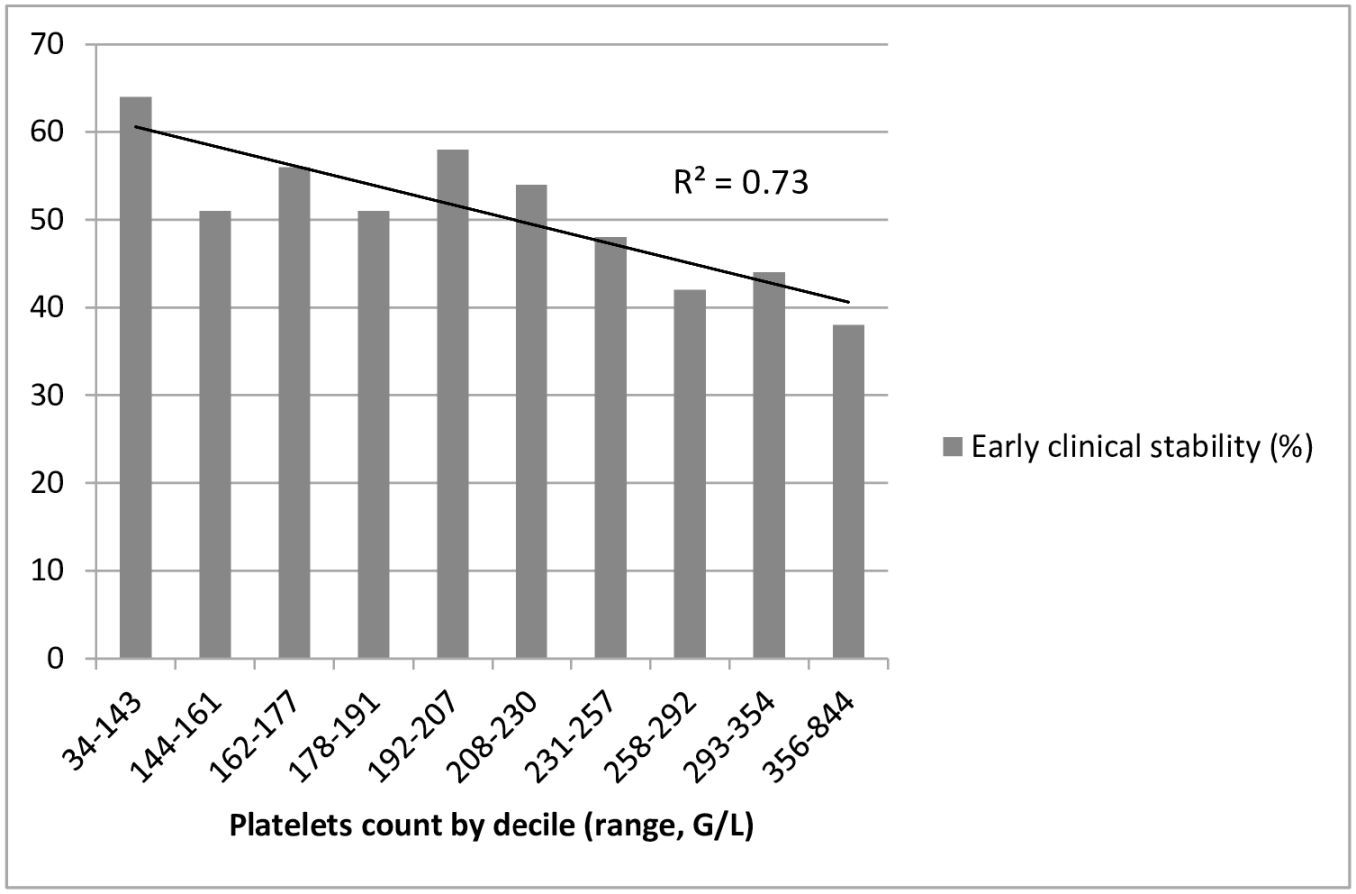
Proportion of patients with early clinical stability by deciles of platelet count.

We performed additional analyses to explore the unexpected association between a lower platelet count and early clinical stability. A lower platelet count remained strongly associated with the outcome when adjusting for PSI category or CURB-65 scores (OR for each increment of 10 G/L of the platelet count: 0.97, p = 0.004). Distribution of platelets by deciles suggested a linear association with early clinical stability, without any J-curve pattern (Fig 1).

### Association between early clinical stability and other outcomes

Early clinical stability was strongly associated with fewer admissions to the intensive care unit and death up to 90 days after admission. Length of stay was shorter for patients with early clinical stability (median length of stay 6 vs. 10 days). There was no association between early clinical stability and complicated pleural effusion or risk of readmission after discharge. (Table 3)

**Table 3:** Association between early clinical stability and other outcomes.

	Stability (N = 293)	No stability (N = 287)	P value
**Intensive care unit admission**	8 (2.7)	23 (8.0)	0.005
**Complicated pleural effusion**≠	7 (2.4)	15 (5.2)	0.07
**Length of stay, median days (IQR)**	6.0 (4.0–10.0)	10.0 (7.0–15.0)	<0.001
**In-hospital death**	2 (0.7)	13 (4.5)	0.004
**30-day death**	4 (1.4)	20 (7.0)	0.001
**90-day death**	10 (3.4)	34 (11.9)	<0.001
**30-day readmission**	14 (4.8)	18 (6.3)	0.43

All data are provided as n (%) if not stated otherwise

^≠^ Need for thoracic drainage or surgery

### Treatment modifications in patients with and without early clinical stability

The initial antibiotic treatment was changed after 72 hours in 28 (9.6%) patients with and 37 (12.9%) patients without early clinical stability (p = 0.20). The most common reported reasons for changing the initial treatment were decision of the physician in charge of the patient (without a specific reason documented), isolation of a resistant pathogen, persisting fever, and admission to the intensive care unit. The frequency of these reasons did not differ significantly between the two categories. There were fewer extrapulmonary infections (0 vs 4) and resistant pathogens (4 vs 7) in patients with, compared with patients without, early clinical stability. However, absolute numbers were small.

## Discussion

Half of patients with moderately severe CAP reached clinical stability within 3 days. Reaching this endpoint was associated with a shorter hospital stay and a better prognosis.

Among the characteristics independently associated with early clinical stability in the first multivariate model, younger age, lower respiratory rate, and absence of hypoxemia are also components of the validated severity scores PSI or CURB-65.[18,19] Although originally developed to predict 30-day mortality, both severity scores were also strongly associated with absence of early clinical stability in case of higher values, a finding also described by others.[20,21]

Respiratory failure is the most frequent direct cause of death in CAP,[22] and correlates of respiratory impairment are closely associated with prognosis. Thorough measurement of respiratory rate and oxygenation indices are essential when assessing a patient early in the course of CAP. Respiratory rate is easily obtained and is included in most clinical prediction rules in CAP, [18,19,23] but its measurement is frequently omitted in clinical routine.[24,25] Interestingly, temperature, a highly valued sign of infection, was not associated with the outcome.

In our second model, younger age, a lower number of co-morbid conditions, and a lower number of symptoms or signs at presentation were predictive of early clinical stability. Age and co-morbid conditions have been associated with a higher risk of failure in previous work.[8,14,16] Conversely, in a prospective study including 1145 patients, Menendez et al. found that dyspnoea, confusion, pleural effusion, multilobar pneumonia, PSI categories IV-V vs. I-III, and lack of adherence to the guidelines for treatment of CAP, but not age, were independent predictors of longer time to clinical stability.[21] It is unclear if vital signs or results of biological tests were included in the analysis. In another trial including only patients with severe CAP, Hoogewerf et al. found that altered mental state, acidosis and lower arterial partial pressure of oxygen were independently associated with early clinical failure.[26] Differences between these two studies and the present work include definitions of clinical stability as well as the populations included.

In both our multivariate models, a lower platelet count was the unique biological parameter independently associated with early clinical stability. Thrombocytosis has been associated with worse outcomes in CAP in previous studies, both in adults and children.[27,28,29,30] In adults, a higher platelet count has been independently associated with higher mortality, higher rate of pulmonary complications, and a longer length of stay.[27,30] Mortality followed a J-shaped curve, with the lowest mortality in the range of 100–250 G/L. Platelets are involved in the host response to infection,[31] and thrombocytosis might be a surrogate for more severe or prolonged lung inflammation. Alternatively, thrombocytosis can be induced by prolonged hypoxia and is an independent predictor of mortality after acute exacerbations of chronic obstructive pulmonary disease.[32] Although thrombocytopenia is a known severity factor in severe sepsis [33] and in CAP, [27,30] we found a linear association between increasing platelet count and increasing risk of early clinical failure. However, patients with severe pneumonia were excluded from our study, and only 2% (11 / 580) of included patients had a platelet count < 100 G/L. This could have precluded our ability to demonstrate an association between moderate or severe thrombocytopenia and a worse outcome.

Of note, neither procalcitonin nor leukocyte count were predictive of early clinical stability in our trial. Procalcitonin has moderate predictive value for mortality [34], and its association with mortality might be restricted to patients in higher risk classes.[35] In one study, patients with early clinical stability had lower median procalcitonin levels than patients without.[36] Patients included in this study were younger and had higher in-hospital mortality than our patients, which could explain why procalcitonin was not predictive of the outcome in our study.

In the original study, more patients treated with betalactam-macrolide combination therapy than with betalactam monotherapy had reached clinical stability at day seven, although the difference was not statistically significant. Conversely, the empiric antibiotic treatments used had no impact on early clinical stability in this analysis. This finding might suggest that the effect of any antibiotic treatment is not apparent in the early phase of pneumonia, as already hypothesized by others.[6]

Finally, we found that early clinical stability was strongly associated with important clinical and health-economic outcomes, including reduced length of hospital stay and mortality, confirming previous findings.[15,20] Early clinical stability could hence become a valid early endpoint in clinical trials on CAP, particularly in moderately severe disease where mortality is low.[3]

The best management of patients without early clinical stability remains unsettled. In particular, clinicians must choose between repeating the diagnostic work-up, with or without broadening of the empiric antibiotic coverage, and watchful waiting. Patients with advanced age or co-morbid conditions may need more time to recover from pneumonia despite adequate treatment, due to lower physiological reserve. However, the worse prognosis of patients without early clinical stability, including a higher risk of death or admission to the intensive care unit, suggests that close monitoring and consideration for repeat investigations or modification of the treatment are warranted in these patients. In our study, the prevalence of extrapulmonary infections or resistant pathogens was low among patients without early clinical stability; however, new investigations in case of lack of stability were not mandated by the protocol but made at the discretion of the clinicians. Close scrutiny should be given to the evolution of signs of respiratory failure, including repeat measurement of respiratory rate and oxygenation indices, to differentiate slow-resolving from progressive pneumonia. The best response in this common clinical dilemma should be explored in a randomized clinical trial.

Our study has significant strengths: it was nested in a prospective study, which implied rigorous and systematic diagnosis definitions, data collection, and outcomes adjudication. Vital signs were measured according to a pre-established protocol. Patients included were mostly elderly people, with a high burden of co-morbid diseases, hence representative of patients hospitalized for CAP. There were few missing data, and no patient was lost to follow-up. Some limitations must be acknowledged. Firstly, only patients with moderately severe CAP were included. Thus, our results cannot be generalized to patients with severe CAP. In addition, some clinical characteristics associated with a worse prognosis, such as multilobar CAP or low blood pressure, were rare in our population and were therefore not included in the multivariate models. Secondly, the association of a lower platelet count with early clinical stability was unexpected and could be a spurious finding. However, similar results have been described by others, and a pathophysiological mechanism related to inflammation may be provided, making this association plausible. Thirdly, we did not measure the evolution of inflammatory biomarkers during the study. As kinetics of biomarkers is indicative of prognosis in CAP,[36,37] such measurements could inform decisions when facing lack of early clinical stability in a patient. Finally, the results of our first multivariable model could suffer from incorporation bias, as the same variables were used as predictors and as part of the outcome definition. Hence, the results of this first model should be considered with caution, even if their face validity seems good.

## Conclusion

We found that younger age, less co-morbid conditions, lower signs or symptoms burden at admission, lower respiratory rate, absence of hypoxemia, and lower platelet count were associated with a higher probability of reaching clinical stability at day 3. Almost half of the patients failed to reach early clinical stability, which was associated with a worse prognosis. Thorough evaluation of respiratory rate and oxygen saturation are simple means that help detect patients at risk for an adverse outcome. Future research should differentiate among the patients failing to reach early clinical stability between those warranting new investigations, modification of the antibiotic treatment, or use of adjunct therapy and those that can be safely observed for slower resolution of vital signs alteration.

## Supporting Information

S1 Table Minimal data set of the study. (XLSX)
